# Interleukin-1β and Interleukin-6 Signaling Differentially Regulate ABC Transporter Activity and Amyloid-β Handling in Primary Porcine Brain Endothelial Cells

**DOI:** 10.3390/cells15141273

**Published:** 2026-07-15

**Authors:** Ahmad H. Razmi, Jeffrey I. Penny

**Affiliations:** Division of Pharmacy and Optometry, School of Health Sciences, Faculty of Biology, Medicine and Health, The University of Manchester, Manchester M13 9PL, UK; ahmad.razmi@manchester.ac.uk

**Keywords:** blood–brain barrier, ABC transporters, ABCB1, ABCG2, ABCC5, amyloid-β, interleukin-6, trans-signaling, neuroinflammation, brain endothelial cells

## Abstract

**Highlights:**

**What are the main findings?**

- Inflammatory cytokines differentially regulate ABCB1, ABCG2, and ABCC5 transporter activity in primary brain endothelial cells in a time- and signaling-pathway-dependent manner.
- IL-6 trans-signaling induces delayed and sustained increases in ABCB1 and ABCC5 activity, whereas classical (cis) IL-6 signaling produces earlier and more transient effects.

**What are the implications of the main findings?**

- Cytokine-mediated modulation of ABC transporters is associated with reduced intracellular amyloid-β accumulation in primary porcine brain endothelial cells, suggesting altered endothelial amyloid-β handling under inflammatory conditions.
- These findings identify inflammatory signaling, particularly IL-6 trans-signaling, as a regulator of endothelial transport processes relevant to Alzheimer’s disease.

**Abstract:**

Impaired amyloid-β clearance at the blood–brain barrier (BBB) contributes to Alzheimer’s disease (AD), yet the regulation of endothelial transport processes under neuroinflammatory conditions remains incompletely understood. Here, we investigated the time-dependent effects of interleukin-1β (IL-1β), interleukin-6 (IL-6) classical signaling directly via the membrane-bound IL-6 receptor, and IL-6 trans-signaling via the IL-6 /soluble IL-6 receptor complex (IL-6/sIL-6r) on ATP-binding cassette (ABC) transporter activity and expression in primary porcine brain endothelial cells (PBECs), and assessed intracellular accumulation of amyloid-β_(1–42)_. Cytokine exposure did not affect PBEC viability. IL-1β induced a robust, time-dependent increase in ABCB1 activity and expression, whereas IL-6 produced a transient enhancement that was not sustained at 72 h. In contrast, IL-6 trans-signaling elicited a delayed but sustained increase in ABCB1 function and expression. IL-1β increased ABCG2 activity and expression at early time points, while IL-6 and IL-6/sIL-6r had minimal effects. Notably, IL-1β and IL-6 trans-signaling increased ABCC5 activity without detectable changes in protein expression. Cytokine-induced transporter modulation was associated with reduced intracellular amyloid-β accumulation. Pharmacological inhibition of ABC transporters increased intracellular amyloid-β levels, supporting a role for these transporters in endothelial amyloid-β handling. However, as transendothelial amyloid-β transport was not directly assessed, these findings should be interpreted as changes in intracellular amyloid-β accumulation rather than direct evidence of altered BBB clearance. These findings demonstrate signaling- and time-dependent regulation of BBB ABC transporters and reveal distinct effects of IL-6 classical signaling and IL-6 trans-signaling on endothelial transporter regulation. Collectively, these results highlight signaling context as an important determinant of BBB transport responses under neuroinflammatory conditions.

## 1. Introduction

Alzheimer’s disease (AD) is characterized by progressive cognitive decline and the accumulation of amyloid-β (Aβ) peptides in the brain parenchyma and cerebral vasculature. Increasing evidence indicates that impaired clearance of Aβ, rather than its overproduction, is a major contributor to sporadic AD [1,2,3]. The blood–brain barrier (BBB) plays a central role in maintaining amyloid-β homeostasis by regulating its transport between the brain and systemic circulation [4].

Amyloid-β transport across the BBB is mediated by multiple complementary systems, including ATP-binding cassette (ABC) efflux transporters and receptor-mediated pathways. Among these, ABCB1 (P-glycoprotein) and ABCG2 (breast cancer resistance protein) are well-established contributors to amyloid-β efflux, whereas ABCC5 has more recently been implicated but remains comparatively understudied [5,6,7,8]. In parallel, low-density lipoprotein receptor-related protein-1 (LRP1) mediates receptor-dependent amyloid-β transport across the BBB and represents a key complementary clearance pathway [4]. Consistent with this, reduced expression or activity of BBB amyloid-β clearance pathways, particularly ABCB1/P-glycoprotein, has been associated with increased cerebral amyloid-β burden in aging and AD [9,10]. However, how these pathways are regulated under pathological conditions remains incompletely defined.

Neuroinflammation is an early and sustained feature of AD and is characterized by activation of glial cells and the release of pro-inflammatory cytokines, including interleukin-1β (IL-1β) and interleukin-6 (IL-6) [11,12,13,14]. These cytokines are known to modulate BBB function; however, their reported effects on BBB ABC transporter regulation remain inconsistent across studies, likely reflecting differences in species, endothelial models, cytokine concentration, and exposure duration [15,16,17]. In addition, most investigations have focused on ABCB1 alone, with comparatively limited evaluation of ABCG2 and minimal data available for ABCC5 in brain endothelial systems.

A further layer of complexity arises from IL-6 signaling, which occurs via two distinct pathways: classical (cis) signaling through the membrane-bound IL-6 receptor and trans-signaling mediated by the IL-6/soluble IL-6 receptor complex (IL-6/sIL-6r), the latter often associated with pro-inflammatory responses [18,19]. Despite the recognized importance of IL-6 in chronic neuroinflammation, the specific impact of IL-6 trans-signaling on BBB transport processes has not been systematically examined [20,21]. Moreover, the time-dependent effects of cytokine exposure and the coordinated regulation of multiple ABC transporters under these conditions remain poorly understood.

Primary porcine brain endothelial cells (PBECs) provide a physiologically relevant in vitro model of the BBB, exhibiting high barrier integrity and robust expression of key BBB transport systems comparable to those observed in human brain endothelium [22,23]. Accordingly, this model enables controlled investigation of cytokine-mediated modulation of endothelial transport processes.

In the present study, we investigated the time-dependent effects of IL-1β, IL-6 (classical signaling), and IL-6/sIL-6r (trans-signaling) on the activity and protein expression of ABCB1, ABCG2, and ABCC5 in PBECs. We further assessed whether cytokine-induced modulation of transporter activity is associated with altered intracellular accumulation of fluorescently labeled amyloid-β_(1–42)_. By directly comparing classical (cis) IL-6 signaling and trans IL-6 signaling pathways, this work provides insight into how inflammatory cues regulate endothelial transport processes relevant to amyloid-β handling at the BBB.

## 2. Materials and Methods

### 2.1. Reagents

Recombinant porcine interleukin-1β (IL-1β) and the recombinant human interleukin-6/soluble IL-6 receptor complex (IL-6/sIL-6r) were obtained from R&D Systems (Abingdon, UK). Recombinant porcine interleukin-6 (IL-6), Bradford reagent, Cellytic™ M lysis buffer, and protease inhibitor cocktail were purchased from Bio-Rad (Watford, UK). Calcein-AM, Hoechst 33342, puromycin dihydrochloride, and Neutral Red were obtained from Sigma-Aldrich (Poole, UK), CMFDA from Abcam (Cambridge, UK), and fluorescently labeled amyloid-β (FAM-Aβ_(1–42)_) from AnaSpec (Fremont, CA, USA). Verapamil hydrochloride (ABCB1 inhibitor), MK571 (ABCC-family inhibitor), and KO143 (ABCG2 inhibitor) were purchased from Tocris Bioscience (Bristol, UK). Rat tail collagen type I and human fibronectin were obtained from BD Biosciences (Oxford, UK). Primary antibodies used in this study included rabbit monoclonal anti-ABCB1 (Abcam, Cambridge, UK; ab170904), rabbit monoclonal anti-ABCG2 (Abcam, Cambridge, UK; ab108312), rabbit polyclonal anti-ABCC5 (Proteintech, Rosemont, IL, USA; 13266-1-AP), and mouse monoclonal anti-β-actin (Sigma-Aldrich, Poole, UK; A5441). HRP-conjugated goat anti-rabbit and goat anti-mouse secondary antibodies were purchased from Santa Cruz Biotechnology (Dallas, TX, USA). All other reagents were of analytical grade unless otherwise stated.

### 2.2. Isolation and Culture of Primary Porcine Brain Endothelial Cells

Primary porcine brain endothelial cells (PBECs) were isolated from Landrace × White pig brains obtained from a local abattoir using a capillary enrichment and enzymatic digestion protocol adapted from the work of Rubin et al. [24], and modified as previously described for PBEC culture systems [22,23]. Briefly, brains were transported to the laboratory in ice-cold DMEM supplemented with penicillin/streptomycin. Meninges and white matter were removed under sterile conditions, and grey matter was finely minced and mechanically homogenized using a glass Dounce homogenizer. The homogenate was sequentially filtered through nylon meshes (150 μm followed by 60 μm) to enrich for microvessels. Capillary fragments retained on the 60 μm mesh were enzymatically digested in M199 medium supplemented with collagenase type III, DNase I, and trypsin at 37 °C with gentle agitation. Following digestion, microvessels were collected by low-speed centrifugation, washed to remove debris and residual enzymes, and resuspended in endothelial growth medium. Isolated PBECs were either plated immediately onto collagen/fibronectin-coated culture ware or cryopreserved in FBS:DMSO (9:1) for subsequent use.

Culture ware was coated with rat tail collagen type I (100 µg/mL in 20 mM acetic acid, 2 h, room temperature), followed by human fibronectin (7.5 µg/mL, overnight, 4 °C). PBECs were seeded at a density of approximately 1.0 × 10^5^ cells/cm^2^ and maintained in low-glucose, phenol red-free DMEM supplemented with plasma-derived serum, heparin, L-glutamine, and penicillin/streptomycin. To remove contaminating cells, PBECs were treated with puromycin (4 µg/mL, 48 h), after which the medium was replaced with a 1:1 mixture of PBEC growth medium and astrocyte-conditioned medium (ACM). Cells were subsequently seeded into pre-coated 96-well plates (approximately 25,000 cells per well) for transporter activity assays, while PBECs maintained in pre-coated 6-well plates were used directly for Western blot analysis once confluent (typically 4 days post-seeding).

This isolation and purification approach, including puromycin selection and astrocyte-conditioned medium, has been previously shown to yield highly enriched brain microvascular endothelial cell cultures exhibiting characteristic BBB properties.

### 2.3. Astrocyte-Conditioned Medium

CTX-TNA2 rat astrocytes (ATCC, Manassas, VA, USA; passages 12–20) were maintained in DMEM supplemented with 10% (*v*/*v*) fetal bovine serum and penicillin/streptomycin at 37 °C in 5% CO_2_. For generation of astrocyte-conditioned medium (ACM), confluent cultures were incubated in fresh growth medium for 48 h. ACM was collected, filtered (0.22 µm sterile filter), and stored at −20 °C. ACM was mixed 1:1 with PBEC growth medium prior to use for maintenance of PBEC cultures.

### 2.4. Cytokine Treatments

PBEC monolayers were treated with IL-1β, IL-6, or IL-6/sIL-6r at a final concentration of 10 ng/mL for 24, 48, or 72 h, as indicated in the figure legends. Treatments were performed using a staggered design such that all conditions, including untreated controls, were assayed simultaneously at a common experimental endpoint.

Prior to cytokine exposure, monolayers were equilibrated in phenol red-free, low-glucose DMEM supplemented with L-glutamine. Control monolayers received medium containing an equivalent volume of PBS (vehicle) under identical conditions.

### 2.5. Cell Viability Assay

Cell viability following cytokine exposure (0.5–10 ng/mL; 72 h) was assessed using the Neutral Red uptake assay adapted from Repetto et al. [25]. PBEC monolayers cultured in collagen/fibronectin-coated 96-well plates were incubated with Neutral Red working solution (final concentration 4 µg/mL in complete DMEM) for 2 h at 37 °C. Cells were then washed twice with ice-cold PBS, and intracellular dye was extracted with 150 µL per well of destain solution (1% (*v*/*v*) acetic acid, 49% (*v*/*v*) distilled water, and 50% (*v*/*v*) ethanol). Plates were gently agitated for 10–15 min at room temperature to ensure complete dye solubilization. Absorbance was measured at 540 nm using a multimode plate reader, and cell viability was expressed as a percentage of control wells.

### 2.6. ABC Transporter Functional Activity Assays

Following cytokine treatment, confluent PBEC monolayers cultured in collagen/fibronectin-coated 96-well plates were used to assess the functional activity of ABC transporters. For all assays, cells were equilibrated in phenol red-free, low-glucose DMEM at 37 °C for 30 min prior to substrate loading.

#### 2.6.1. ABCB1 Activity

ABCB1 functional activity was assessed by measuring intracellular accumulation of calcein generated from calcein-AM. Cells were incubated with calcein-AM (0.5 µM, 30 min, 37 °C, protected from light), followed by washing with ice-cold PBS to remove extracellular substrate. Intracellular fluorescence was measured using a Hidex Sense multimode plate reader (excitation 485 nm; emission 530 nm). Lower intracellular fluorescence values reflect reduced intracellular accumulation of calcein.

#### 2.6.2. ABCG2 Activity

ABCG2 functional activity was assessed using the Hoechst 33342 accumulation assay. Cells were incubated with Hoechst 33342 (5 µM, 30 min, 37 °C, protected from light), followed by washing with ice-cold PBS. Intracellular fluorescence was measured using a Hidex Sense multimode plate reader (excitation 350 nm; emission 461 nm). Intracellular fluorescence reflects the accumulation of Hoechst 33342 within cells.

#### 2.6.3. ABCC5 Activity

ABCC5 functional activity was assessed using conversion of 5-chloromethylfluorescein diacetate (CMFDA) to the fluorescent glutathione conjugate GS-MF. Cells were incubated with CMFDA (4 µM, 30 min, 37 °C, protected from light), washed with ice-cold PBS, and intracellular fluorescence was measured (excitation 492 nm; emission 517 nm). Intracellular fluorescence reflects accumulation of GS-MF within cells.

### 2.7. Intracellular Amyloid-β Accumulation Following Cytokine Treatment

To assess the effect of cytokine exposure on amyloid-β handling, PBEC monolayers cultured in collagen/fibronectin-coated 96-well plates were treated with IL-1β, IL-6, or IL-6/sIL-6r (10 ng/mL) for 24, 48, or 72 h. Following treatment, cells were washed once with pre-warmed PBS and equilibrated in phenol red-free, low-glucose DMEM supplemented with L-glutamine for 1 h at 37 °C.

FAM-labeled amyloid-β_(1–42)_ was added at a final concentration of 1 µM. The peptide was prepared according to the manufacturer’s instructions immediately prior to use and was not subjected to any additional oligomerization procedure. Consequently, experiments were performed using freshly prepared peptide preparations expected to consist predominantly of monomeric Aβ species. Cells were incubated for 2 h at 37 °C protected from light. Following incubation, monolayers were washed five times with ice-cold PBS to remove extracellular peptide and terminate further uptake or efflux.

Intracellular fluorescence was measured immediately using a Hidex Sense multimode plate reader (excitation 494 nm; emission 521 nm). A standard curve of FAM-amyloid-β_(1–42)_ was generated in parallel to enable conversion of fluorescence intensity to absolute peptide levels. Data processing and normalization are described in Section 2.9.

### 2.8. Transporter Inhibition Studies and Amyloid-β Accumulation

To evaluate the contribution of ABC transporters to amyloid-β handling, PBEC monolayers were pre-treated with transporter inhibitors prior to incubation with FAM-labeled amyloid-β_(1–42)_. Cells were incubated with verapamil (10 µM; ABCB1 inhibitor), KO143 (0.5 µM; ABCG2 inhibitor), MK571 (25 µM; ABCC-family inhibitor), or a combination of all three inhibitors for 30 min at 37 °C.

Following inhibitor pre-treatment, FAM-amyloid-β_(1–42)_ was added at a final concentration of 1 µM, and cells were incubated for a further 2 h at 37 °C protected from light. For temperature-dependence studies, parallel experiments were performed at 4 °C following addition of FAM-amyloid-β_(1–42)_.

After incubation, monolayers were washed five times with ice-cold PBS to remove extracellular peptide and terminate transport processes. Intracellular fluorescence was measured using a Hidex Sense multimode plate reader (excitation 494 nm; emission 521 nm). Fluorescence values were converted to absolute peptide levels using a FAM-amyloid-β_(1–42)_ standard curve. Data processing and normalization are described in Section 2.9.

### 2.9. Protein Quantification and Normalization

Total protein content in PBEC monolayers was determined using the Bradford assay. Cells were incubated in ice-cold PBS (160 µL per well), followed by addition of Bradford reagent (40 µL per well). Plates were gently agitated and incubated at room temperature for 5 min to allow for color development. Absorbance was measured at 595 nm using a Hidex Sense multimode plate reader (Hidex, Turku, Finland).

Protein concentrations were calculated from a standard curve generated using bovine serum albumin (BSA) standards (0–2 mg/mL), prepared and measured under identical conditions.

For ABC transporter functional assays, fluorescence values were normalized to total protein content per well and expressed relative to control conditions as a percentage.

For amyloid-β accumulation assays, fluorescence values were first converted to absolute peptide levels using a FAM-amyloid-β_(1–42)_ standard curve. These values were then normalized to total protein content and expressed relative to control conditions as a percentage.

### 2.10. Western Blotting

Following cytokine treatment (24–72 h), PBECs were lysed directly in culture wells using Cellytic™ M lysis buffer supplemented with a protease inhibitor cocktail. Cells were collected by scraping and lysates transferred to microcentrifuge tubes. Total protein concentration was determined using the Bradford assay, and samples were stored at −80 °C until analysis.

For Western blotting, protein samples were mixed with Laemmli sample buffer and equal amounts of total protein were loaded per lane (50 µg for ABCB1 and ABCG2; 100 µg for ABCC5). Proteins were separated by SDS–PAGE using 10% polyacrylamide gels for ABCB1 and ABCG2 and 8% gels for ABCC5, run at a constant voltage of 120 V. Proteins were transferred to PVDF membranes using a wet transfer system at 400 mA for 2 h at 4 °C.

Membranes were blocked in 5% (*w*/*v*) non-fat dry milk in Tris-buffered saline containing 0.1% (*v*/*v*) Tween-20 (TBS-T) for 1 h at room temperature, followed by overnight incubation at 4 °C with primary antibodies against ABCB1 (1:500), ABCG2 (1:500), ABCC5 (1:250), or β-actin (1:25,000). After washing in TBS-T, membranes were incubated with HRP-conjugated goat anti-rabbit or goat anti-mouse secondary antibodies (1:5000) for 1 h at room temperature.

Protein bands were visualized using enhanced chemiluminescence and imaged using a Bio-Rad ChemiDoc™ system. Densitometric analysis was performed using ImageJ software (version 1.53k; National Institutes of Health, Bethesda, MD, USA). Target protein expression was normalized to β-actin and expressed relative to control conditions.

### 2.11. Statistical Analysis

Data are presented as mean ± SD from at least three independent biological experiments. The exact number of biological replicates (*n*) for each experiment is indicated in the corresponding figure legends. Each independent experiment comprised multiple technical replicates, which were averaged prior to statistical analysis. Statistical analyses were performed using biological replicate means.

Statistical analyses were performed using GraphPad Prism (version 9.0; GraphPad software, San Diego, CA, USA). For comparisons involving multiple treatment groups at a single time point, one-way analysis of variance (ANOVA) was used, followed by Dunnett’s post hoc test for comparisons with a single control group. Assumptions of normality and homogeneity of variance were assessed using the Shapiro–Wilk test and Levene’s test, respectively. A *p* value < 0.05 was considered statistically significant.

### 2.12. Ethical Approval

Porcine brain tissue was obtained from a local abattoir, and no animals were specifically sacrificed for this study. Ethical approval for the use of porcine brain tissue to isolate primary porcine brain endothelial cells was granted by the University of Manchester Committee for Ethical Review.

## 3. Results

### 3.1. Cytokine Treatments Do Not Affect PBEC Viability

To exclude cytotoxic effects of cytokine exposure, PBEC viability was assessed following treatment with IL-1β, IL-6, or IL-6/sIL-6r (0.5–10 ng/mL) for 72 h using the Neutral Red uptake assay.

**Figure 1:**
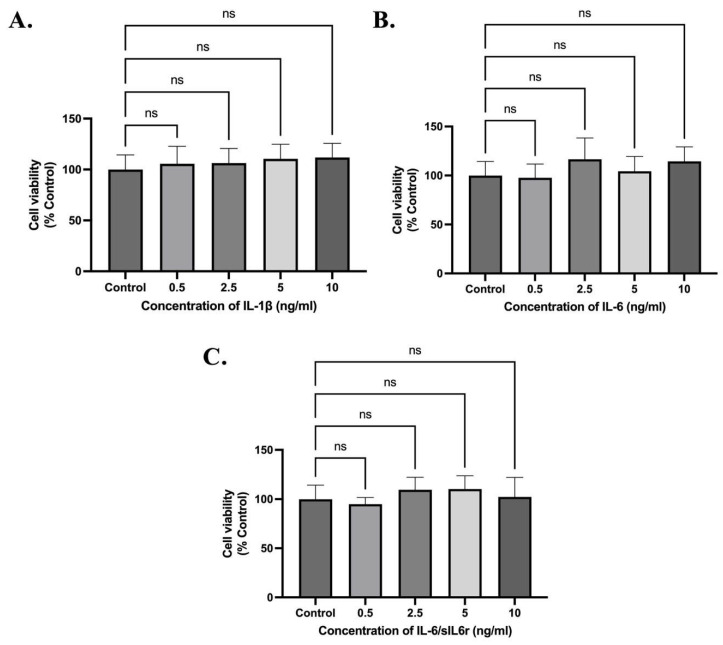
Effects of IL-1β, IL-6, and IL-6/sIL-6r on PBEC viability. Primary porcine brain endothelial cells (PBECs) were treated for 72 h with increasing concentrations (0.5–10 ng/mL) of cytokines. (**A**) IL-1β; (**B**) IL-6; (**C**) IL-6/sIL-6r. Cell viability was assessed using the Neutral Red uptake assay. Data are presented as mean ± SD from three independent experiments (*n* = 3), each performed with four technical replicates, and are expressed as a percentage of control conditions. Statistical significance was determined using one-way ANOVA followed by Dunnett’s post hoc test; ns, not significant.

None of the cytokine treatments significantly affected PBEC viability across the concentration range tested (Figure 1A–C). Cell viability remained comparable to control conditions following exposure to IL-1β (Figure 1A), IL-6 (Figure 1B), and IL-6/sIL-6r (Figure 1C).

These data indicate that the cytokine concentrations used in this study do not induce overt cytotoxicity under the experimental conditions employed. Based on these findings, a concentration of 10 ng/mL was selected for subsequent experiments.

### 3.2. Cytokines Differentially Regulate ABCB1 Functional Activity in PBECs

To determine whether inflammatory cytokines modulate ABCB1 functional activity, PBECs were treated with IL-1β, IL-6, or IL-6/sIL-6r for 24, 48, or 72 h, and transporter activity was assessed using the calcein-AM accumulation assay.

**Figure 2:**
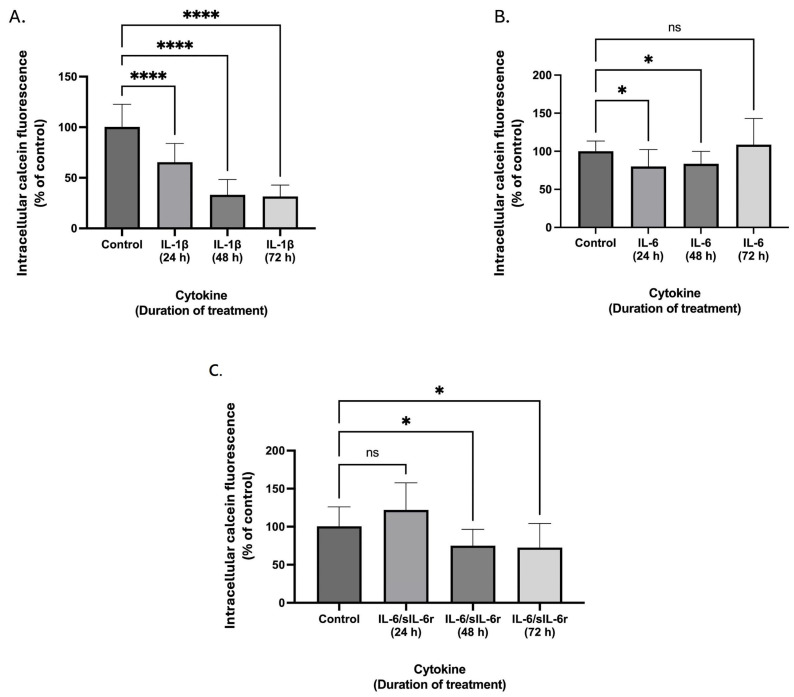
Time-dependent effects of IL-1β, IL-6, and IL-6/sIL-6r on ABCB1 functional activity in PBECs. Primary porcine brain endothelial cells (PBECs) were treated with cytokines (10 ng/mL) for 24, 48, or 72 h using a staggered treatment design. (**A**) IL-1β; (**B**) IL-6; (**C**) IL-6/sIL-6r. ABCB1 functional activity was assessed using the calcein-AM accumulation assay. Intracellular fluorescence was normalized to total protein content and expressed as a percentage of control conditions. Data are presented as mean ± SD from four independent experiments (*n* = 4), each performed with four technical replicates. Statistical significance was determined using one-way ANOVA followed by Dunnett’s post hoc test; * *p* < 0.05, **** *p* < 0.0001; ns, not significant.

IL-1β treatment resulted in a clear, time-dependent reduction in intracellular calcein fluorescence compared with control conditions (Figure 2A). Significant reductions were observed at 24 h, with further decreases at 48 h and 72 h.

IL-6 treatment produced a more modest and transient effect (Figure 2B). Intracellular calcein fluorescence was reduced at 24 h and 48 h; however, no significant difference was observed at 72 h compared with control conditions.

In contrast, IL-6/sIL-6r treatment exhibited a delayed response (Figure 2C). No significant change was observed at 24 h, whereas intracellular calcein fluorescence was significantly reduced at 48 h and 72 h.

Overall, these data demonstrate cytokine- and time-dependent modulation of ABCB1 functional activity in PBECs, with distinct temporal profiles observed for IL-1β, IL-6, and IL-6 trans-signaling.

### 3.3. Cytokines Differentially Regulate ABCG2 and ABCC5 Functional Activity in PBECs

To determine whether inflammatory cytokines regulate additional ABC transporters, the effects of IL-1β, IL-6, and IL-6/sIL-6r on ABCG2 and ABCC5 functional activity were assessed following 24, 48, and 72 h treatment.

**Figure 3:**
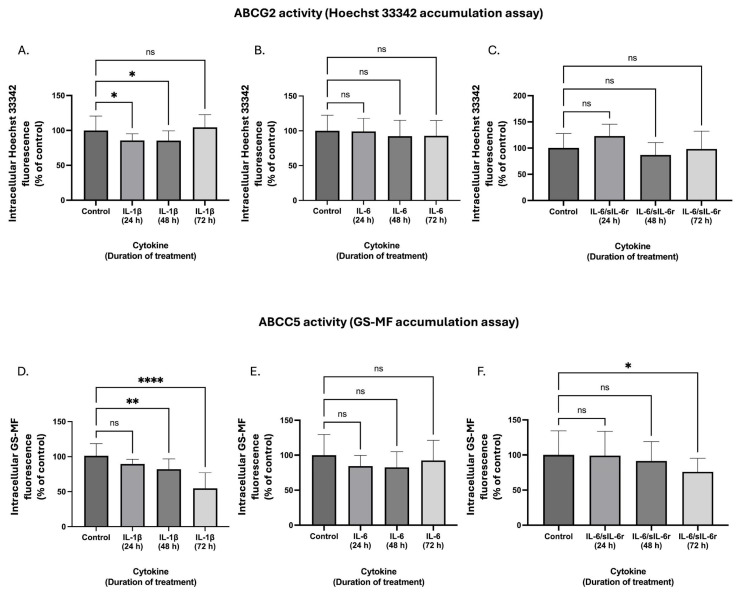
Differential effects of IL-1β, IL-6, and IL-6/sIL-6r on ABCG2 and ABCC5 functional activity in PBECs. Primary porcine brain endothelial cells (PBECs) were treated with cytokines (10 ng/mL) for 24, 48, or 72 h using a staggered treatment design. (**A**–**C**) ABCG2 functional activity assessed using the Hoechst 33342 accumulation assay following treatment with (**A**) IL-1β, (**B**) IL-6, or (**C**) IL-6/sIL-6r. (**D**–**F**) ABCC5 functional activity assessed using the GS-MF accumulation assay following treatment with (**D**) IL-1β, (**E**) IL-6, or (**F**) IL-6/sIL-6r. Intracellular fluorescence was normalized to total protein content and expressed as a percentage of control conditions. Data are presented as mean ± SD from four independent experiments (*n* = 4), each performed with four technical replicates. Statistical significance was determined using one-way ANOVA followed by Dunnett’s post hoc test; * *p* < 0.05, ** *p* < 0.01, **** *p* < 0.0001; ns, not significant.

IL-1β treatment resulted in a modest but significant reduction in intracellular Hoechst 33342 fluorescence at 24 h, with a similar trend observed at 48 h (Figure 3A). No significant difference was detected at 72 h compared with control conditions. In contrast, IL-6 and IL-6/sIL-6r treatment did not significantly alter intracellular Hoechst 33342 fluorescence at any time point examined (Figure 3B,C).

In contrast to ABCG2, ABCC5 functional activity exhibited a distinct temporal response to cytokine exposure. IL-1β treatment resulted in a time-dependent reduction in intracellular GS-MF fluorescence, reaching statistical significance at 48 h and becoming more pronounced at 72 h (Figure 3D). IL-6 treatment did not significantly affect GS-MF fluorescence at any time point (Figure 3E). Notably, IL-6/sIL-6r treatment significantly reduced GS-MF fluorescence at 72 h, whereas earlier time points were not significantly different from control conditions (Figure 3F).

Overall, these data demonstrate transporter- and cytokine-specific modulation of intracellular substrate accumulation, with IL-1β affecting both ABCG2 and ABCC5-associated readouts, whereas IL-6 trans-signaling selectively influences ABCC5 at later time points.

### 3.4. Cytokine Treatment Differentially Modulates ABC Transporter Protein Expression in PBECs

To determine whether cytokine-induced changes in transporter activity were associated with altered protein expression, ABCB1, ABCG2, and ABCC5 levels were assessed following treatment with IL-1β, IL-6, or IL-6/sIL-6r for 24, 48, and 72 h.

**Figure 4:**
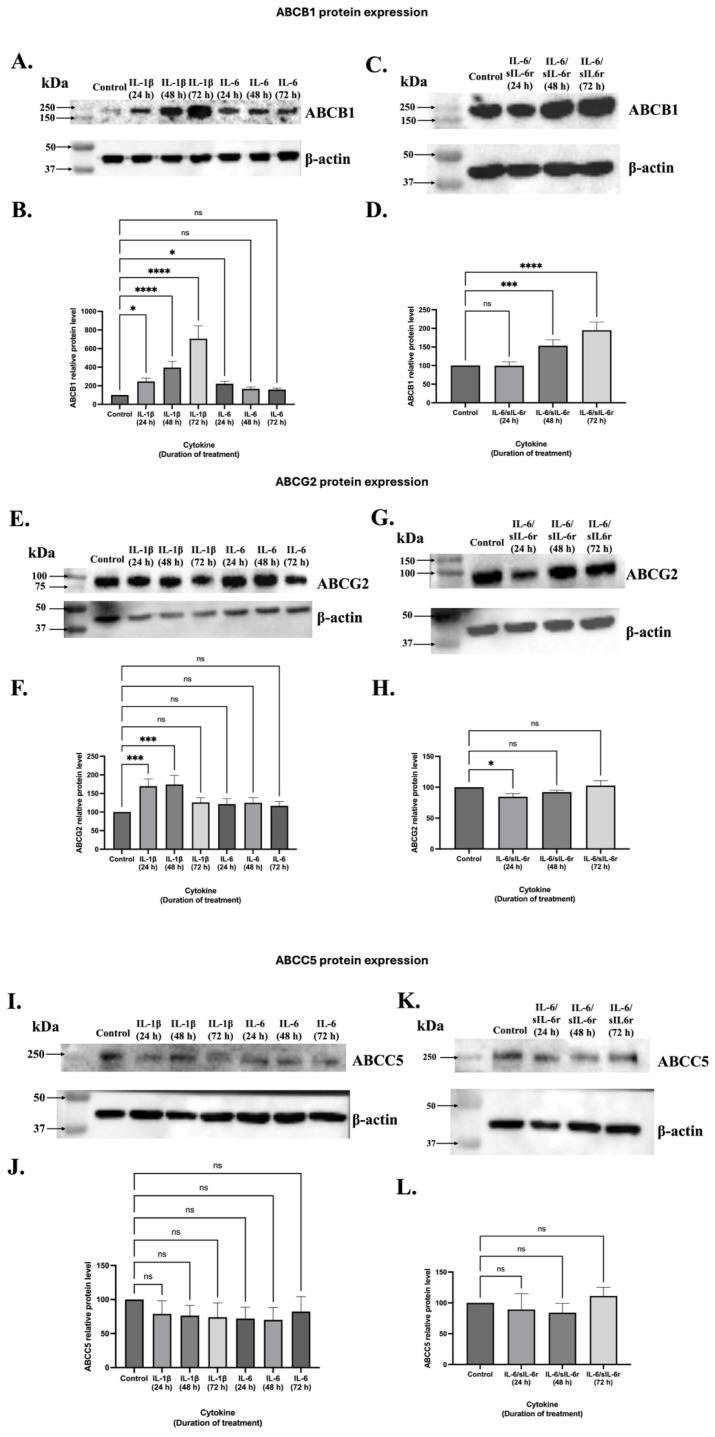
Effects of IL-1β, IL-6, and IL-6/sIL-6r on ABC transporter protein expression in PBECs. Primary porcine brain endothelial cells (PBECs) were treated with cytokines (10 ng/mL) for 24, 48, or 72 h using a staggered treatment design, with all conditions harvested simultaneously at a common experimental endpoint (72 h post-treatment). (**A**–**D**) ABCB1 protein expression: (**A**,**B**) IL-1β and IL-6; (**C**,**D**) IL-6/sIL-6r. (**E**–**H**) ABCG2 protein expression: (**E**,**F**) IL-1β and IL-6; (**G**,**H**) IL-6/sIL-6r. (**I**–**L**) ABCC5 protein expression: (**I**,**J**) IL-1β and IL-6; (**K**,**L**) IL-6/sIL-6r. Representative immunoblots and corresponding densitometric analyses are shown. Protein expression was normalized to β-actin and expressed as a percentage relative to control conditions. Data are presented as mean ± SD from three independent experiments (*n* = 3). Statistical significance was determined using one-way ANOVA followed by Dunnett’s post hoc test; * *p* < 0.05, *** *p* < 0.001, **** *p* < 0.0001; ns, not significant.

IL-1β treatment resulted in a time-dependent increase in ABCB1 protein expression (Figure 4A,B), with significantly elevated levels observed at 24 h, 48 h, and 72 h compared with control conditions. IL-6 treatment produced a transient increase in ABCB1 expression (Figure 4A,B), with a significant elevation observed at 24 h but no significant differences detected at 48 h or 72 h relative to control conditions. In contrast, exposure to IL-6/sIL-6r resulted in a delayed increase in ABCB1 expression (Figure 4C,D), with no significant change at 24 h but significant increases observed at 48 h and 72 h.

For ABCG2, IL-1β treatment increased protein expression at 24 h and 48 h, with no significant difference observed at 72 h (Figure 4E–H). Neither IL-6 nor IL-6/sIL-6r treatment significantly altered ABCG2 expression across the time course examined.

In contrast, ABCC5 protein expression was not significantly affected by cytokine treatment at any time point (Figure 4I–L). Although some variability was observed between samples, no consistent changes in ABCC5 expression were detected.

Overall, these data indicate that cytokine-dependent modulation of transporter activity is accompanied by selective changes in protein expression for ABCB1 and ABCG2, whereas ABCC5 functional modulation occurs in the absence of detectable changes in total protein levels.

### 3.5. Cytokine Treatment Reduces Intracellular Amyloid-β Accumulation in PBECs

To determine whether cytokine-induced changes in transporter activity are associated with altered amyloid-β handling, intracellular accumulation of FAM-labeled amyloid-β_(1–42)_ was assessed following treatment with IL-1β, IL-6, or IL-6/sIL-6r for 24, 48, or 72 h.

**Figure 5:**
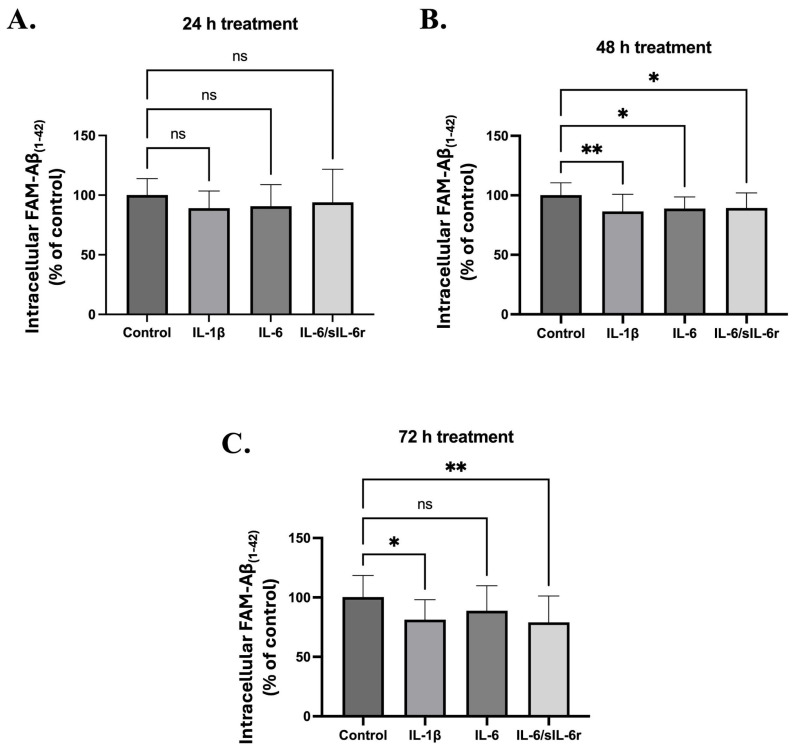
Effects of IL-1β, IL-6, and IL-6/sIL-6r on intracellular amyloid-β accumulation in PBECs. Primary porcine brain endothelial cells (PBECs) were treated with cytokines (10 ng/mL) for (**A**) 24 h, **(B**) 48 h, or (**C**) 72 h. Intracellular accumulation of FAM-labeled amyloid-β_(1–42)_ was measured following a 2 h incubation period. Amyloid-β levels were normalized to total protein content and expressed as a percentage of control conditions. Data are presented as mean ± SD from three independent experiments (*n* = 3), each performed with four technical replicates. Statistical significance was determined using one-way ANOVA followed by Dunnett’s post hoc test; * *p* < 0.05, ** *p* < 0.01; ns, not significant.

No significant differences in intracellular amyloid-β levels were observed following 24 h cytokine treatment compared with control conditions (Figure 5A). In contrast, all cytokine treatments significantly reduced intracellular amyloid-β accumulation after 48 h exposure (Figure 5B), with IL-1β producing the most pronounced reduction and IL-6 and IL-6/sIL-6r inducing more modest decreases.

At 72 h, intracellular amyloid-β levels remained significantly reduced following IL-1β and IL-6/sIL-6r treatment, whereas IL-6 treatment did not result in a significant difference compared with control conditions (Figure 5C).

Overall, these data demonstrate time- and cytokine-dependent reductions in intracellular amyloid-β accumulation in PBECs. These changes are consistent with the observed modulation of ABC transporter functional activity; however, additional mechanisms may also contribute to altered amyloid-β handling under inflammatory conditions.

### 3.6. ABC Transporter Inhibition Increases Intracellular Amyloid-β Accumulation in PBECs

To further assess the contribution of ABC transporters to amyloid-β handling in PBECs, the effects of transporter inhibition on intracellular accumulation of FAM-labeled amyloid-β_(1–42)_ were examined.

**Figure 6:**
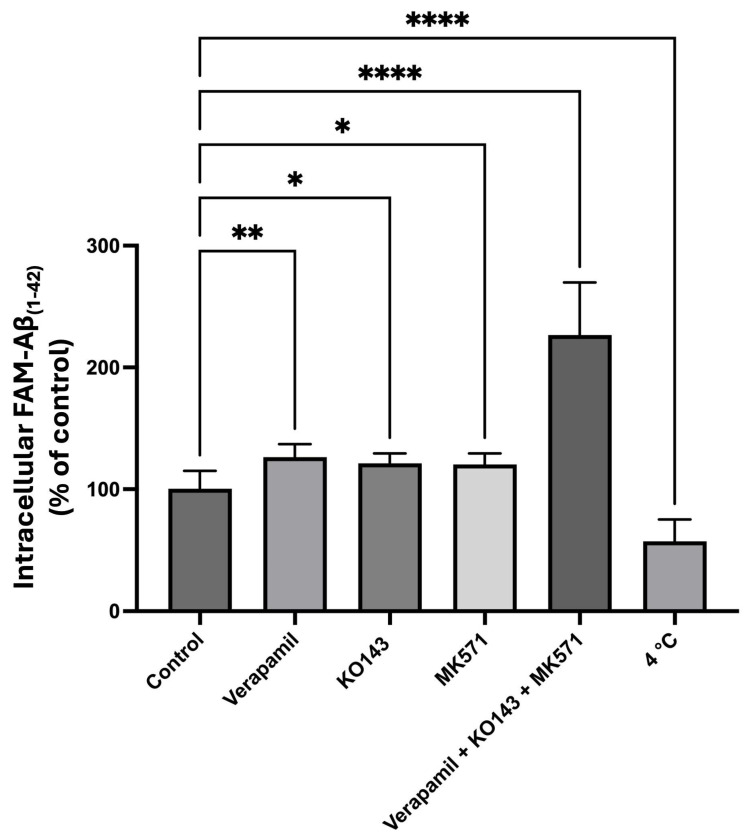
Effects of ABC transporter inhibition and low temperature on intracellular amyloid-β accumulation in PBECs. Primary porcine brain endothelial cells (PBECs) were pre-treated with verapamil (10 µM), KO143 (0.5 µM), MK571 (25 µM), or a combination of all three inhibitors for 30 min prior to incubation with FAM-labeled amyloid-β_(1–42)_ (1 µM) for 2 h. For temperature-dependent experiments, cells were incubated at 4 °C following amyloid-β exposure. Intracellular amyloid-β levels were measured, normalized to total protein content, and expressed as a percentage of control conditions. Data are presented as mean ± SD from four independent experiments (*n* = 4), each performed with at least four technical replicates. Statistical significance was determined using one-way ANOVA followed by Dunnett’s post hoc test; * *p* < 0.05, ** *p* < 0.01, **** *p* < 0.0001.

Pre-treatment with the ABCB1 inhibitor verapamil, the ABCG2 inhibitor KO143, or the ABCC-family inhibitor MK571 resulted in significant increases in intracellular amyloid-β accumulation compared with control conditions (Figure 6). Verapamil produced the largest increase among the individual inhibitors, while KO143 and MK571 induced more modest but significant increases.

Combined inhibition of ABC transporters using verapamil, KO143, and MK571 resulted in a marked increase in intracellular amyloid-β accumulation compared with control conditions, exceeding the effects observed with individual inhibitors.

To assess the contribution of temperature-dependent transport processes, cells were incubated at 4 °C following amyloid-β exposure. This resulted in a significant reduction in intracellular amyloid-β accumulation compared with control conditions.

Collectively, these data support the involvement of ABC transporters and temperature-dependent processes in regulating amyloid-β handling in PBECs.

## 4. Discussion

Neuroinflammation is a central feature of Alzheimer’s disease (AD), with sustained cytokine signaling contributing to disease progression and neurovascular dysfunction [14]. Among key mediators, IL-1β and IL-6 are consistently elevated in AD and are released by activated glial cells and neurons, forming part of a self-amplifying inflammatory network in which amyloid-β (Aβ) further promotes cytokine production [26,27,28,29]. Although anti-inflammatory strategies have not translated into effective therapies [30,31], inflammatory signaling remains a critical regulator of blood–brain barrier (BBB) function and may directly influence Aβ clearance mechanisms [32,33].

Here, we demonstrate that IL-1β, IL-6 classical (cis) signaling, and IL-6 trans-signaling differentially regulate ABC transporter activity and expression in primary porcine brain endothelial cells (PBECs), with downstream consequences for intracellular Aβ handling. Notably, these effects occurred in the absence of cytotoxicity, indicating that cytokine exposure modulates endothelial transport function rather than compromising cell viability.

A principal finding is that cytokine exposure enhances ABC transporter activity in a transporter-, cytokine-, and time-dependent manner. IL-1β induced a robust and sustained increase in ABCB1 activity, accompanied by parallel increases in protein expression, consistent with previous observations in PBECs [34]. In contrast, studies in other endothelial models have reported reduced or unchanged cytokine-mediated ABCB1 regulation [15,16], underscoring the importance of experimental context, including species, model system, cytokine concentration, and exposure duration. The use of 10 ng/mL cytokine concentrations in the present study was based on previous investigations of cytokine-mediated BBB dysfunction and transporter regulation, where concentrations within the low nanogram-per-milliliter range have been widely employed to model inflammatory conditions in vitro [15,16,35]. Although cytokine concentrations within the brain microenvironment are difficult to quantify precisely and may vary substantially according to disease stage and local cellular activity, elevated levels of IL-1β and IL-6 have been reported in AD and other neuroinflammatory disorders [13,36]. Furthermore, exposure to 10 ng/mL cytokines produced robust alterations in transporter activity and expression without affecting PBEC viability under the experimental conditions employed, supporting its suitability for investigating cytokine-mediated regulation of BBB transport processes. Exposure periods of 24, 48, and 72 h were selected to capture both early and delayed cytokine-mediated responses, as changes in transporter activity and expression can develop over timescales ranging from several hours to multiple days following inflammatory stimulation.

IL-6 exerted a transient effect on ABCB1 activity, with increased function observed at 24–48 h but not sustained at 72 h. In contrast, selective activation of IL-6 trans-signaling using the IL-6/sIL-6r complex resulted in a delayed but sustained increase in ABCB1 activity and protein expression. These findings highlight the importance of signaling context and suggest that classical and trans-signaling pathways exert distinct regulatory effects on endothelial efflux capacity. This may partly explain discrepancies across previous studies, where IL-6 has been reported to increase, decrease, or not affect ABCB1 activity depending on the endothelial model, IL-6 receptor availability, and experimental conditions [15,16,20,21,37].

The mechanisms underlying the differential effects of IL-6 classical signaling and IL-6 trans-signaling on BBB transporter regulation remain to be fully elucidated. Both signaling pathways activate gp130-dependent intracellular signaling cascades, including the JAK/STAT3 pathway; however, IL-6 trans-signaling is often associated with more sustained and pro-inflammatory cellular responses than classical IL-6 signaling [18]. In contrast, IL-1β predominantly signals through activation of NF-κB and mitogen-activated protein kinase (MAPK) pathways, both of which have been implicated in the regulation of endothelial transporter expression and function [38,39,40]. The distinct temporal profiles observed in the present study may therefore reflect differential activation and cross-talk between JAK/STAT3, NF-κB, and MAPK signaling pathways. One possible explanation for the transient nature of the IL-6 response is the activation of endogenous negative-feedback mechanisms. Classical IL-6 signaling induces suppressor of cytokine signaling (SOCS) proteins, particularly SOCS3, which can attenuate JAK/STAT signaling and limit the duration of downstream cellular responses [41,42,43]. In addition, receptor internalization and down-regulation following prolonged cytokine exposure may further reduce signaling over time [43]. In contrast, IL-6 trans-signaling has been associated with more sustained inflammatory responses in several cell types and may therefore maintain transporter regulation over longer periods [18,42]. However, these mechanisms were not directly investigated in the current study and remain to be clarified. Future studies will focus on establishing the relative contributions of these signaling pathways to cytokine-mediated regulation of BBB transport processes.

A key finding of the present study is that IL-6 classical signaling and IL-6 trans-signaling produced distinct temporal effects on BBB transporter regulation. Previous studies have typically examined IL-6 as a single inflammatory mediator without distinguishing between classical and trans-signaling pathways [15,16]. The present findings therefore indicate that signaling context is an important determinant of endothelial transporter regulation and suggest that classical and trans-signaling pathways may differentially influence BBB transport function under neuroinflammatory conditions.

Cytokine-dependent regulation extended to additional efflux transporters. IL-1β increased ABCG2 activity and protein expression at early time points, whereas IL-6 classical signaling and IL-6 trans-signaling did not significantly alter ABCG2 functional activity or protein expression across the time course examined. These findings are consistent with previous reports demonstrating model-dependent regulation of ABCG2 by inflammatory cytokines, with IL-1β shown to increase ABCG2 expression in some cellular systems, whereas IL-6 has been reported to exert minimal or inhibitory effects depending on the endothelial context [15,44,45]. In contrast, ABCC5 displayed a distinct regulatory profile. IL-1β and IL-6 trans-signaling increased ABCC5 functional activity at later time points without detectable changes in total protein expression. This apparent dissociation between transporter activity and expression suggests that mechanisms other than altered protein abundance may contribute to ABCC5 regulation. ABC transporters are known to undergo dynamic regulation via altered trafficking to the plasma membrane, changes in membrane localization, and post-translational modifications that influence transport capacity without affecting total protein levels [46,47,48,49]. We therefore hypothesize that the increased ABCC5 activity observed in the present study may reflect enhanced transporter trafficking, membrane localization, or post-translational activation at the cell surface rather than increased expression. However, these mechanisms were not directly assessed and therefore remain to be clarified. Although data on ABCC5 regulation in brain endothelial cells remain limited, inflammatory conditions have been shown to modulate members of the ABCC transporter family in other tissues [50], supporting the concept that ABCC5 is responsive to inflammatory signaling. The present findings therefore provide novel evidence that IL-1β and IL-6 trans-signaling can enhance ABCC5 functional activity in a time-dependent manner independent of changes in total protein expression.

Importantly, cytokine-induced changes in transporter activity were associated with reduced intracellular accumulation of FAM-Aβ_(1–42)_ in a time-dependent manner. No significant effects on intracellular accumulation were observed at 24 h, whereas reductions became apparent at 48–72 h, coinciding with more pronounced and coordinated increases in transporter activity. This temporal relationship likely reflects the complexity of Aβ handling at the BBB, where intracellular levels represent a balance between uptake, intracellular trafficking, and efflux processes mediated by both receptor- and transporter-dependent pathways [4,7]. Consequently, intracellular Aβ measurements provide a net readout of these combined processes rather than a direct measure of efflux alone [6,15,47].

In addition to ABC transporters, receptor-mediated pathways such as low-density lipoprotein receptor-related protein 1 (LRP1) and the receptor for advanced glycation end products (RAGE) play important roles in Aβ trafficking across the BBB. LRP1 facilitates brain-to-blood transport of Aβ and is known to be influenced by neuroinflammatory and neurovascular changes associated with AD [32,33]. In contrast, RAGE contributes to endothelial Aβ uptake and has been implicated in the influx of circulating Aβ into the brain [51]. Therefore, the reduction in intracellular Aβ observed following cytokine exposure may reflect combined contributions from ABC transporter-mediated efflux and receptor-mediated transport pathways. While the present study focused on ABC transporter regulation, the potential involvement of LRP1 and RAGE cannot be excluded. Consistent with this possibility, preliminary unpublished observations from our laboratory indicate that pharmacological inhibition of RAGE significantly reduces intracellular FAM-Aβ_(1–42)_ accumulation in PBECs, supporting a role for receptor-mediated uptake in determining intracellular Aβ levels. Future studies will therefore be required to define the relative contributions of transporter- and receptor-mediated pathways to cytokine-induced alterations in endothelial Aβ handling.

Functional evidence supporting a role for ABC transporters in Aβ handling was provided by pharmacological inhibition experiments. Inhibition of ABCB1, ABCG2, and ABCC transporters increased intracellular accumulation of FAM-Aβ_(1–42)_, with combined inhibition producing a greater effect than individual inhibitors, consistent with additive or cooperative contributions of multiple efflux transporter pathways. Furthermore, reduced Aβ accumulation at 4 °C confirmed temperature dependence, supporting the involvement of active transport processes. Together, these findings support a role for ABC transporters in the regulation of intracellular Aβ levels in PBECs.

While the present findings suggest that inflammatory cytokine signaling may enhance endothelial Aβ handling through increased ABC transporter activity, the overall consequences of sustained neuroinflammation for BBB function are likely to be considerably more complex. For example, prolonged exposure to IL-1β has been reported to disrupt tight junction integrity and increase BBB permeability, potentially compromising barrier function despite enhanced transporter activity [52,53]. Similarly, although IL-6 signaling has been implicated in BBB dysfunction under certain inflammatory conditions, its effects on tight junction proteins and barrier integrity remain less consistent across experimental models [40,54]. Consequently, the net effect of chronic neuroinflammation on BBB-mediated Aβ homeostasis is likely to reflect a balance between potentially beneficial effects on transporter-mediated Aβ handling and detrimental effects on barrier integrity and neurovascular function [14]. Further studies examining both transporter regulation and barrier properties will therefore be required to determine the overall impact of inflammatory signaling on BBB function in AD.

The fluorescence-based accumulation assays employed here provide indirect measures of transporter function, as they reflect intracellular substrate accumulation rather than direct transport kinetics. However, these assays are widely used and produced distinct transporter-associated response profiles in PBECs under the experimental conditions employed [8,55]. The consistency of the functional response patterns observed in this study, together with concordance between functional activity and protein expression for ABCB1 and ABCG2, supports the interpretation that the observed effects predominantly reflect modulation of ABC transporter function.

Several considerations should be noted. First, intracellular Aβ accumulation reflects the combined effects of uptake, intracellular trafficking, degradation, and efflux processes and therefore does not distinguish between these individual components [56]. Consequently, the reductions in intracellular FAM-Aβ_(1–42)_ observed following cytokine exposure should be interpreted as evidence of altered endothelial Aβ handling rather than direct evidence of enhanced BBB Aβ clearance. Furthermore, directional transendothelial Aβ transport was not assessed in the present study, as experiments were performed using PBEC monolayers cultured in conventional multiwell plates rather than Transwell systems. Future studies employing Transwell-based BBB models and measurements of apical-to-basolateral and basolateral-to-apical Aβ transport will be required to determine whether cytokine-induced transporter regulation translates into altered BBB clearance capacity. An additional limitation of the present study is that experiments were performed using PBEC monocultures rather than a multicellular neurovascular unit (NVU) model. While PBECs retain many key BBB characteristics, the model does not incorporate interactions with astrocytes, pericytes, microglia, or neurons, all of which contribute to BBB regulation in vivo [57,58]. Astrocytes are an important source of inflammatory mediators, including IL-1β and IL-6, and can influence endothelial transporter expression and barrier function through secondary signaling mechanisms [59]. Although PBECs were maintained in astrocyte-conditioned medium to support BBB phenotype, the model does not fully reproduce the complex cellular interactions that occur within the AD brain. Consequently, the present findings should be interpreted as direct effects of cytokine exposure on brain endothelial cells rather than a complete representation of neurovascular responses to neuroinflammation. Future studies employing co-culture and multicellular NVU models will be important to determine how endothelial and glial signaling interact to regulate BBB transport processes under inflammatory conditions [57]. In addition, inflammatory cytokines are known to influence BBB integrity through effects on tight junction proteins and paracellular permeability [40,52,53]. Although barrier integrity measurements such as transendothelial electrical resistance (TEER), paracellular permeability, and tight junction protein expression were not evaluated in the present study, future investigations should assess the combined effects of inflammatory signaling on both transporter regulation and barrier function. Although PBECs represent a highly physiologically relevant in vitro BBB model and reproduce many key structural and functional characteristics of the human BBB [57,60], species-specific differences in cytokine signaling, receptor expression, and transporter regulation may influence the translational applicability of these findings [60]. Consequently, validation of the present observations in human BBB models will be important to establish the extent to which these cytokine-mediated effects are conserved across species. Finally, while the present study establishes functional associations between cytokine signaling and transporter regulation, the underlying molecular mechanisms remain to be defined.

Collectively, these findings demonstrate that IL-1β, IL-6 classical signaling, and IL-6 trans-signaling differentially regulate ABC transporter activity and expression at the BBB in a signaling- and time-dependent manner, with corresponding effects on Aβ handling.

## 5. Conclusions

In conclusion, inflammatory cytokines differentially regulate ABC transporter activity and expression in primary porcine brain endothelial cells in a time- and signaling-dependent manner. These changes were associated with altered intracellular accumulation of Aβ_(1–42)_, supporting a functional link between cytokine signaling and BBB transport processes. Importantly, the distinct effects of IL-6 classical signaling and IL-6 trans-signaling identify signaling context as a critical determinant of endothelial transporter regulation. Together, these findings advance our understanding of how neuroinflammation influences BBB transport function and provide a foundation for future studies investigating the interplay between ABC transporters, receptor-mediated pathways such as LRP1 and RAGE, and BBB integrity in the regulation of Aβ homeostasis.

## Data Availability

The original contributions presented in this study are included in this article/Supplementary Materials. Further inquiries can be directed to the corresponding author.

## Supplementary Materials

The following supporting information can be downloaded at: https://www.mdpi.com/article/10.3390/cells15141273/s1, Figure S1: Uncropped Western blot images corresponding to Figure 4.

## Abbreviations

The following abbreviations are used in this manuscript:

ABC	ATP-binding cassette
ABCB1	ATP-binding cassette subfamily B member 1
ABCG2	ATP-binding cassette subfamily G member 2
ABCC5	ATP-binding cassette subfamily C member 5
ACM	Astrocyte-conditioned medium
AD	Alzheimer’s disease
Aβ	Amyloid-β
BBB	Blood–brain barrier
BSA	Bovine serum albumin
CMFDA	5-chloromethylfluorescein diacetate
DMEM	Dulbecco’s modified Eagle medium
DMSO	Dimethyl sulfoxide
FAM-Aβ_(1–42)_	Fluorescein amidite-labeled amyloid-β_(1–42)_
FBS	Fetal bovine serum
GS-MF	Glutathione-methylfluorescein
HRP	Horseradish peroxidase
IL-1β	Interleukin-1β
IL-6	Interleukin-6
IL-6R	Interleukin-6 receptor
IL-6/sIL-6r	Interleukin-6/soluble interleukin-6 receptor complex
LRP1	Low-density lipoprotein receptor-related protein 1
PBECs	Primary porcine brain endothelial cells
PBS	Phosphate-buffered saline
PVDF	Polyvinylidene fluoride
RAGE	Receptor for advanced glycation end-products
SDS-PAGE	Sodium dodecyl sulfate–polyacrylamide gel electrophoresis
sIL-6r	Soluble interleukin-6 receptor
TBS-T	Tris-buffered saline with Tween-20
