# Supplementary material for: Interleukin-1β and Interleukin-6 Signaling Differentially Regulate ABC Transporter Activity and Amyloid-β Handling in Primary Porcine Brain Endothelial Cells
Source: Cells. 2026 Jul 15;15(14):1273. doi: 10.3390/cells15141273 (PMC13406441; doi:10.3390/cells15141273)
Supplement: Supplementary file 1 [file cells-15-01273-s001.zip › cells-4368805-supplementary.pdf]

Supplementary Figure S1. Uncropped Western blot images corresponding to Figure 4.

Full, uncropped Western blot images corresponding to representative blots shown in Figure 4. Membranes were cut prior to probing for ABC transporters and β-actin. Cropped regions used for quantification in the main figures are indicated by red boxes.

Supplementary Figure S1A – Uncropped ABCB1 and β-actin blots (corresponds to Figure 4A)

ABCB1 blot (uncropped)

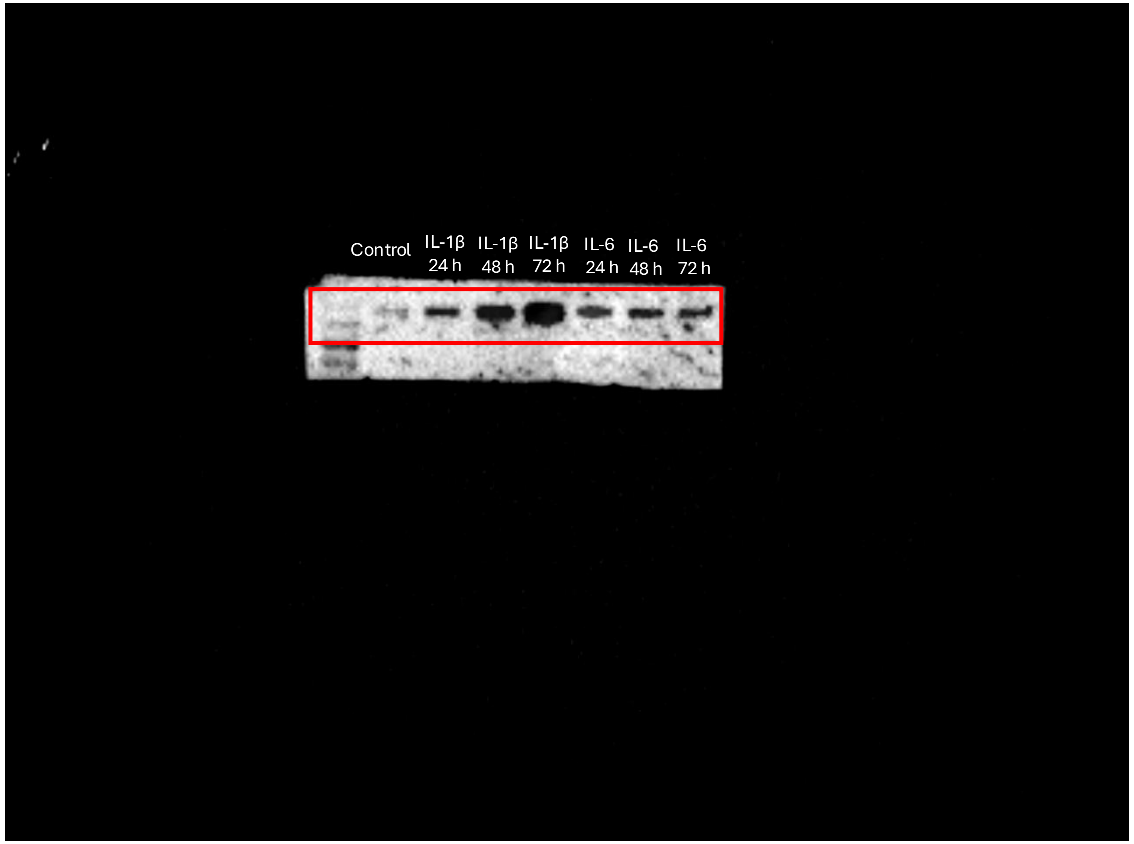

β-actin blot (uncropped)

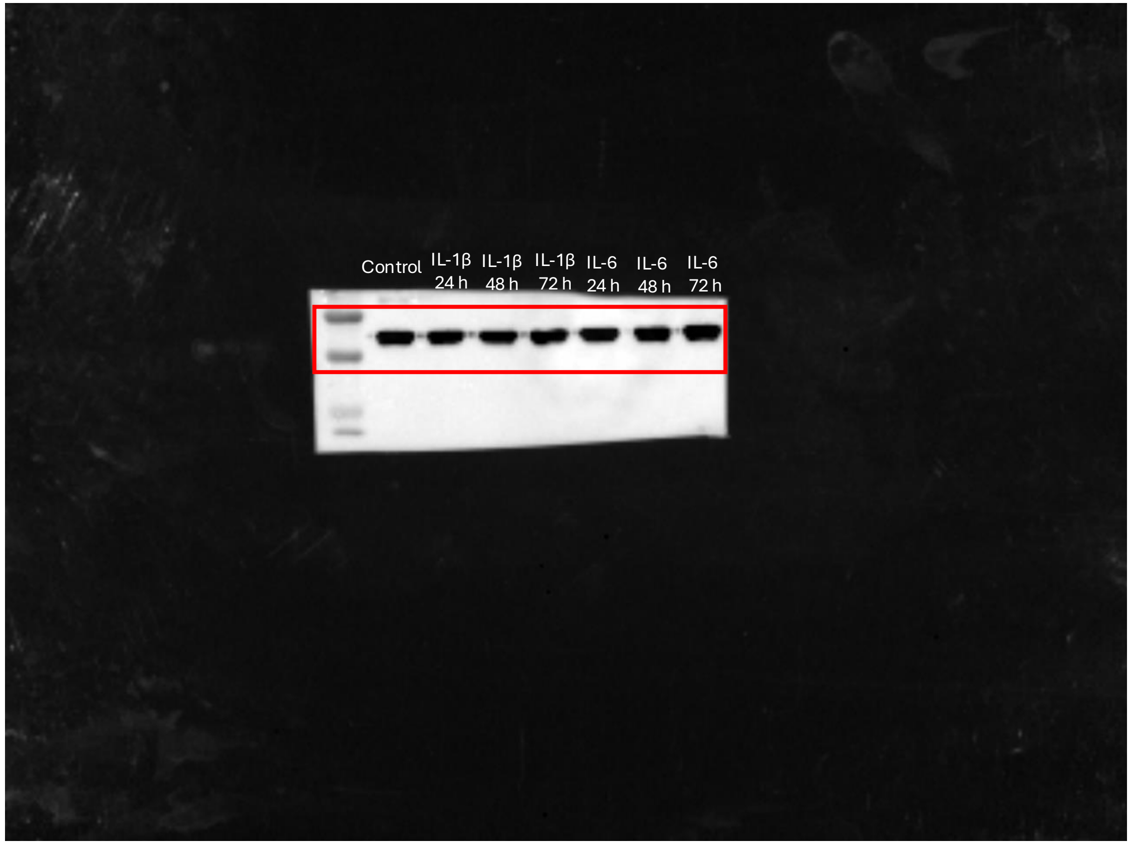

- ABCB1 (uncropped membrane)

- β-actin (corresponding loading control)

Supplementary Figure S1B – Uncropped ABCB1 and β-actin blots (corresponds to Figure 4C)

ABCB1 blot (uncropped)

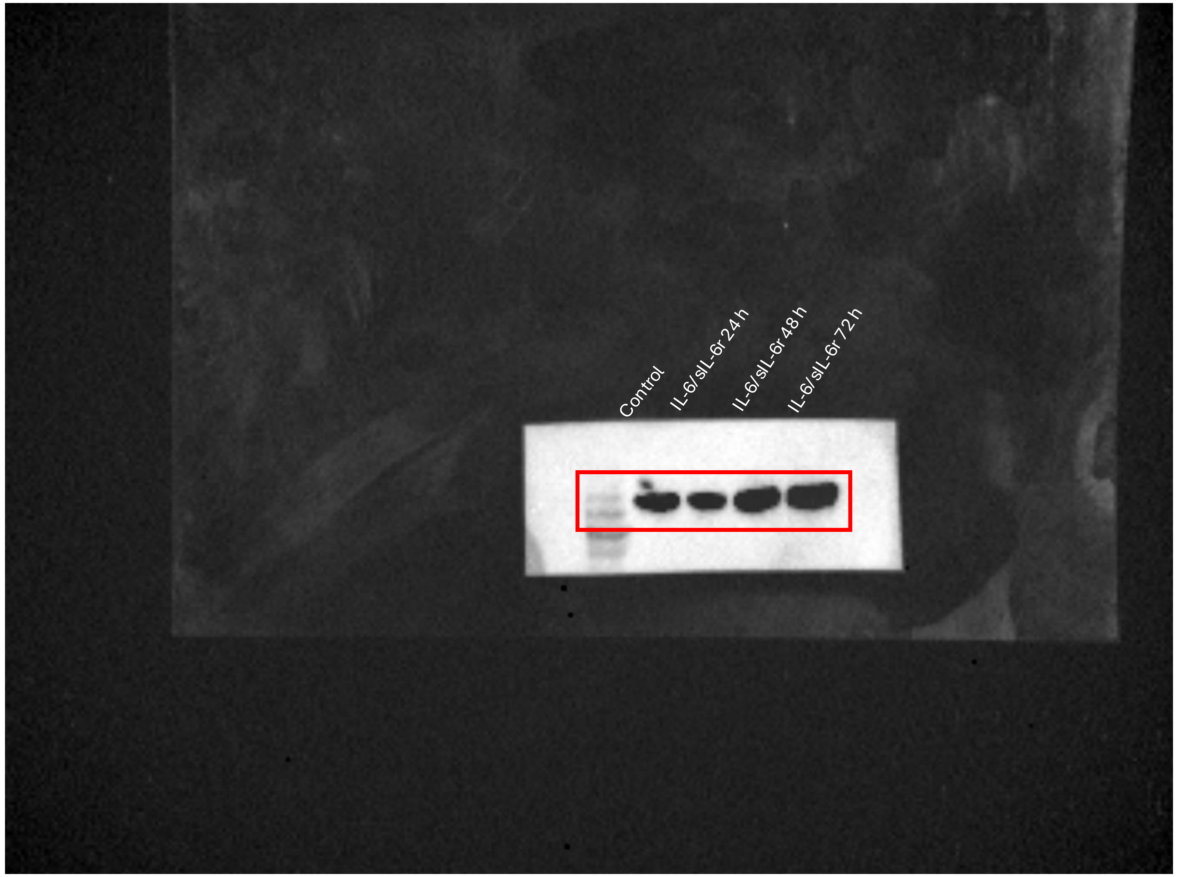

- ABCB1 (uncropped membrane)

β-actin blot (uncropped)

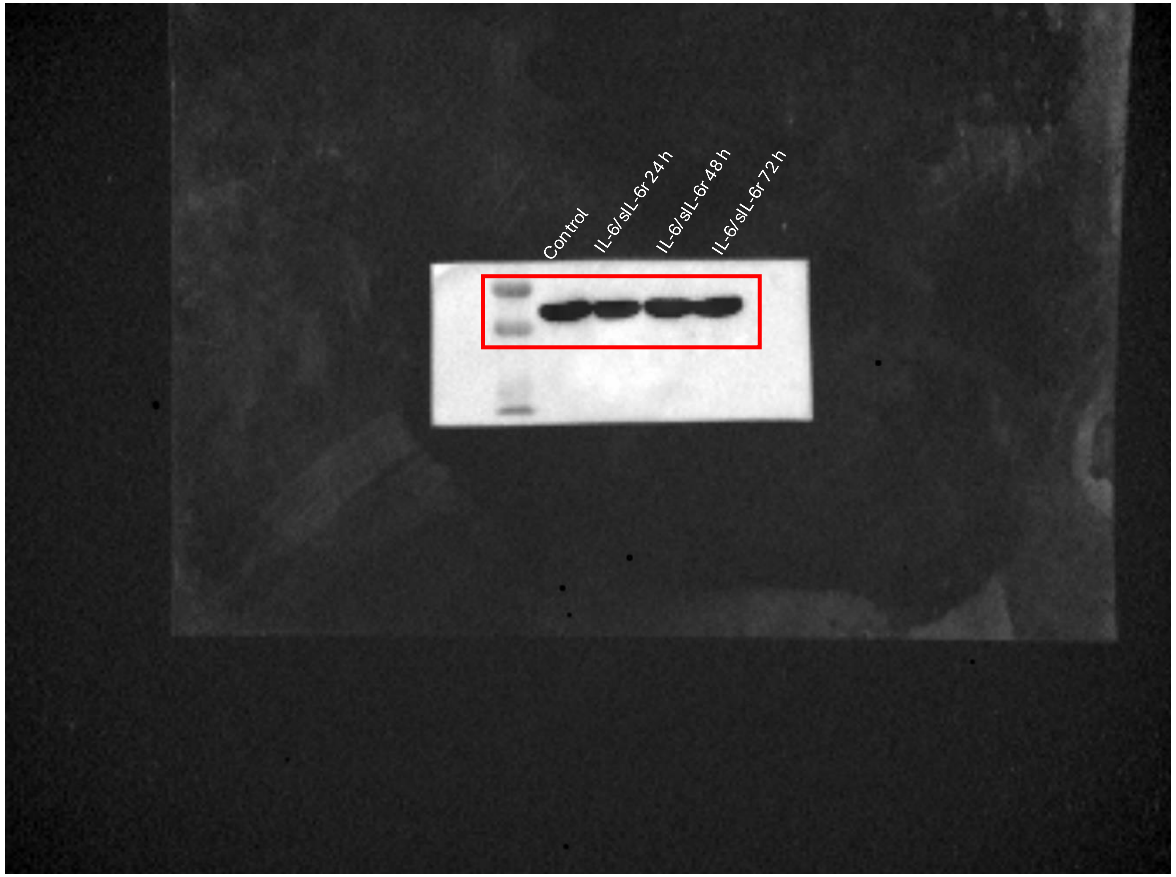

- β-actin (corresponding loading control)

Supplementary Figure S1C – Uncropped ABCG2 and β-actin blots (corresponds to Figure 4E)

ABCG2 blot (uncropped)

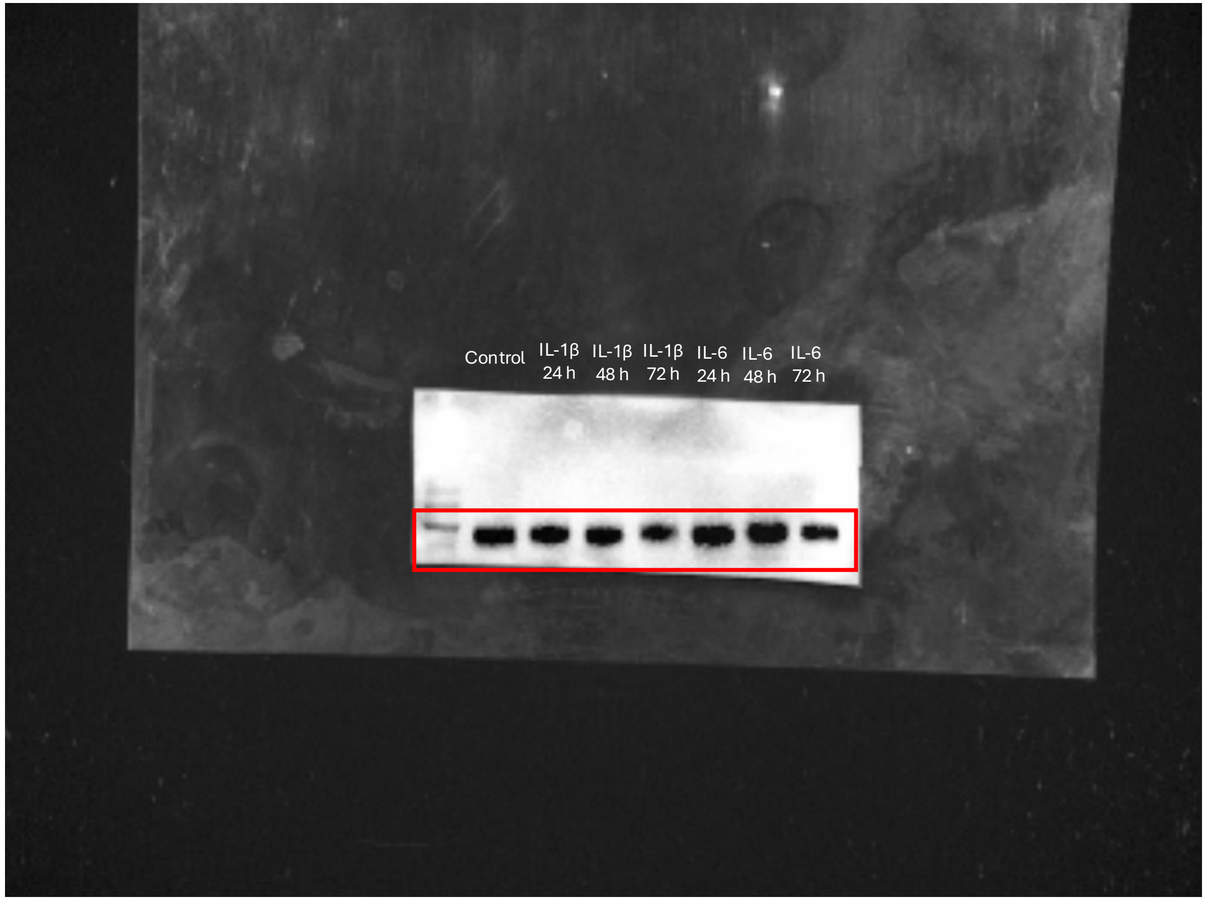

- ABCG2 (uncropped membrane)

β-actin blot (uncropped)

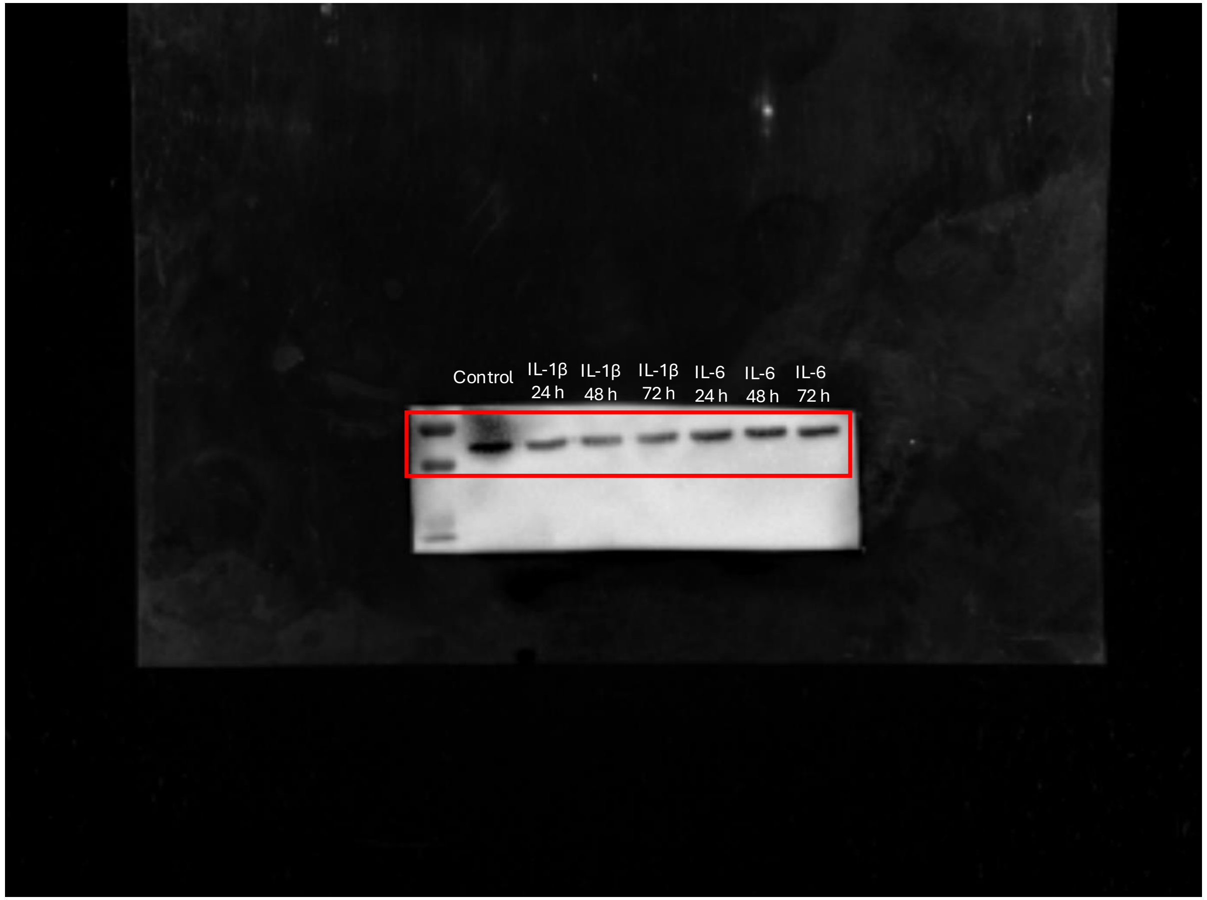

- β-actin (corresponding loading control)

Supplementary Figure S1D – Uncropped ABCG2 and β-actin blots (corresponds to Figure 4G)

ABCG2 blot (uncropped)

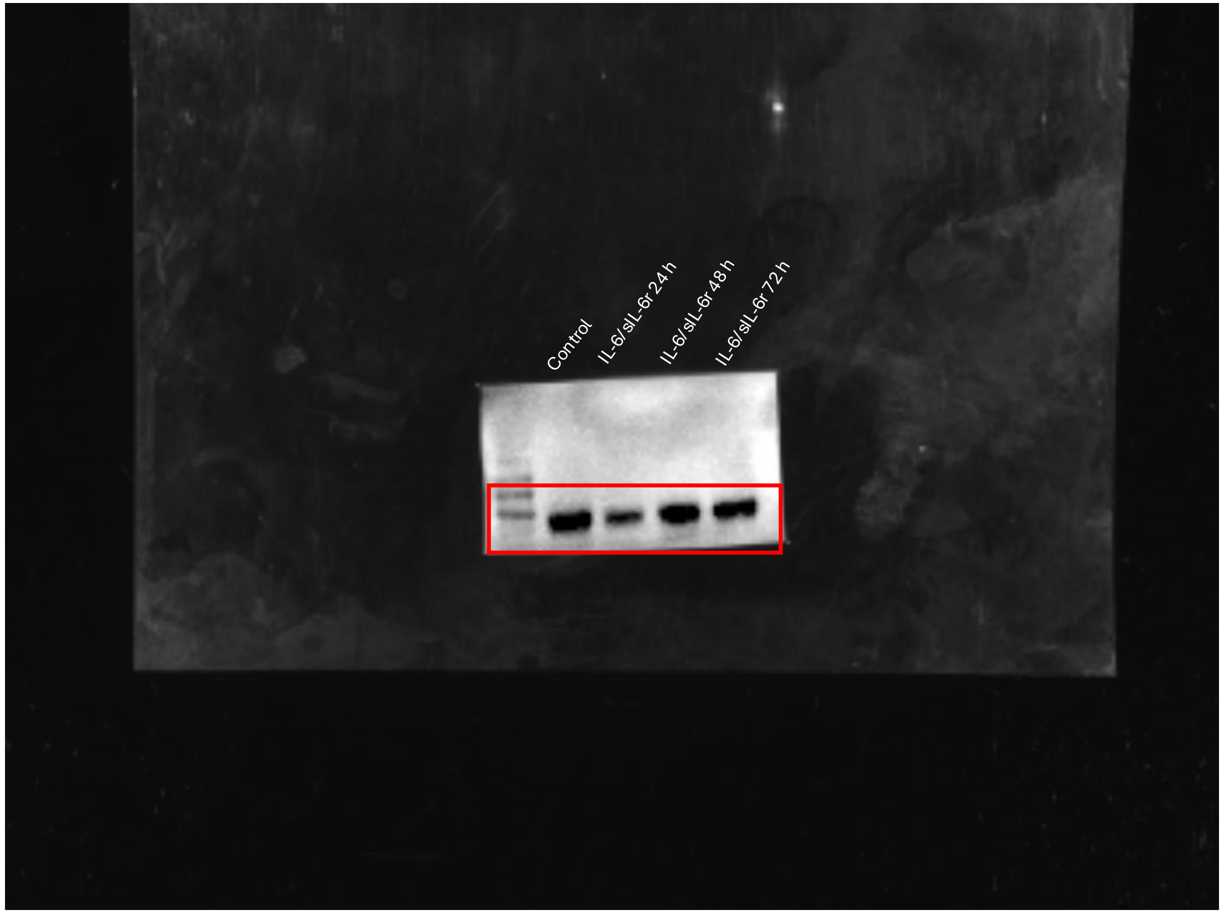

β-actin blot (uncropped)

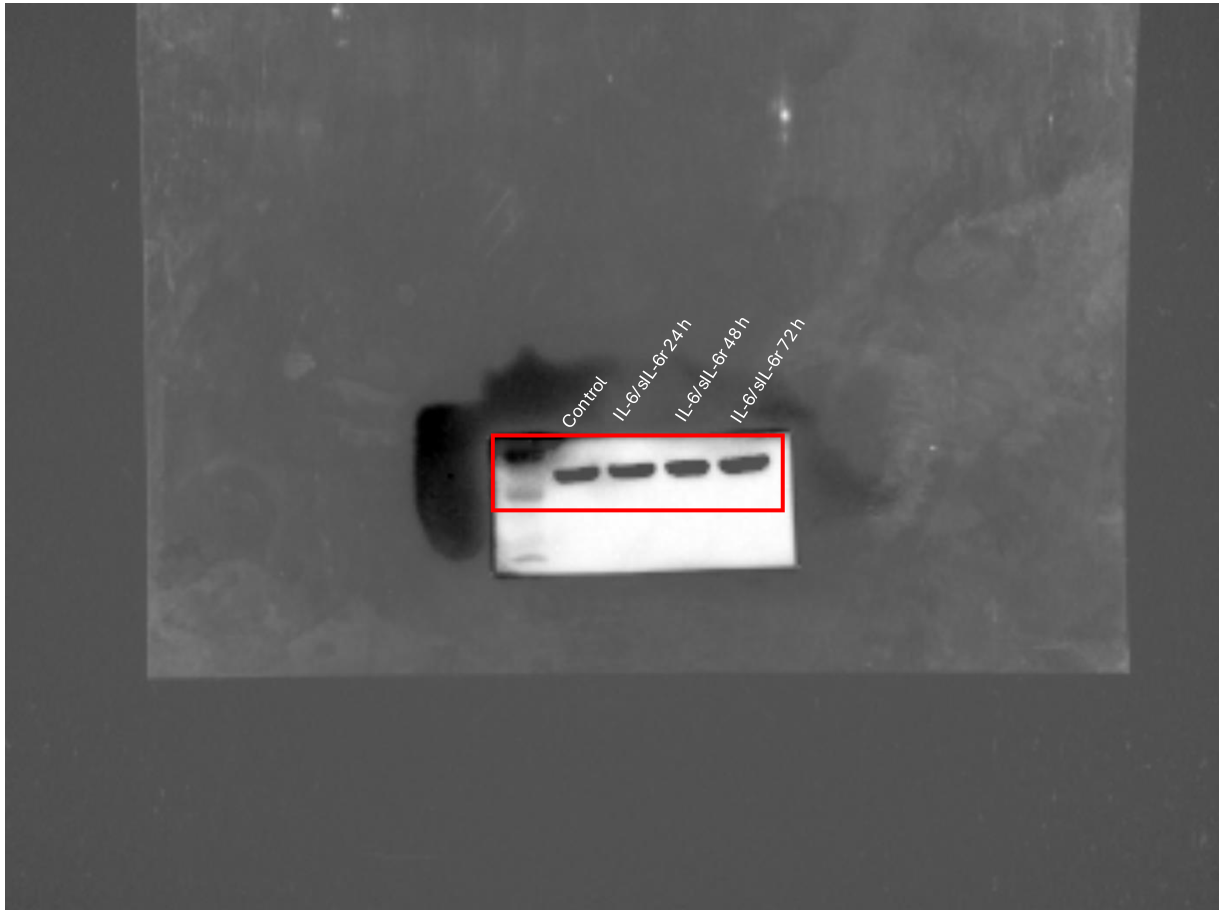

- ABCG2 (uncropped membrane)
- β-actin (corresponding loading control)

Supplementary Figure S1E – Uncropped ABCC5 and β-actin blots (corresponds to Figure 4I)

ABCC5 blot (uncropped)

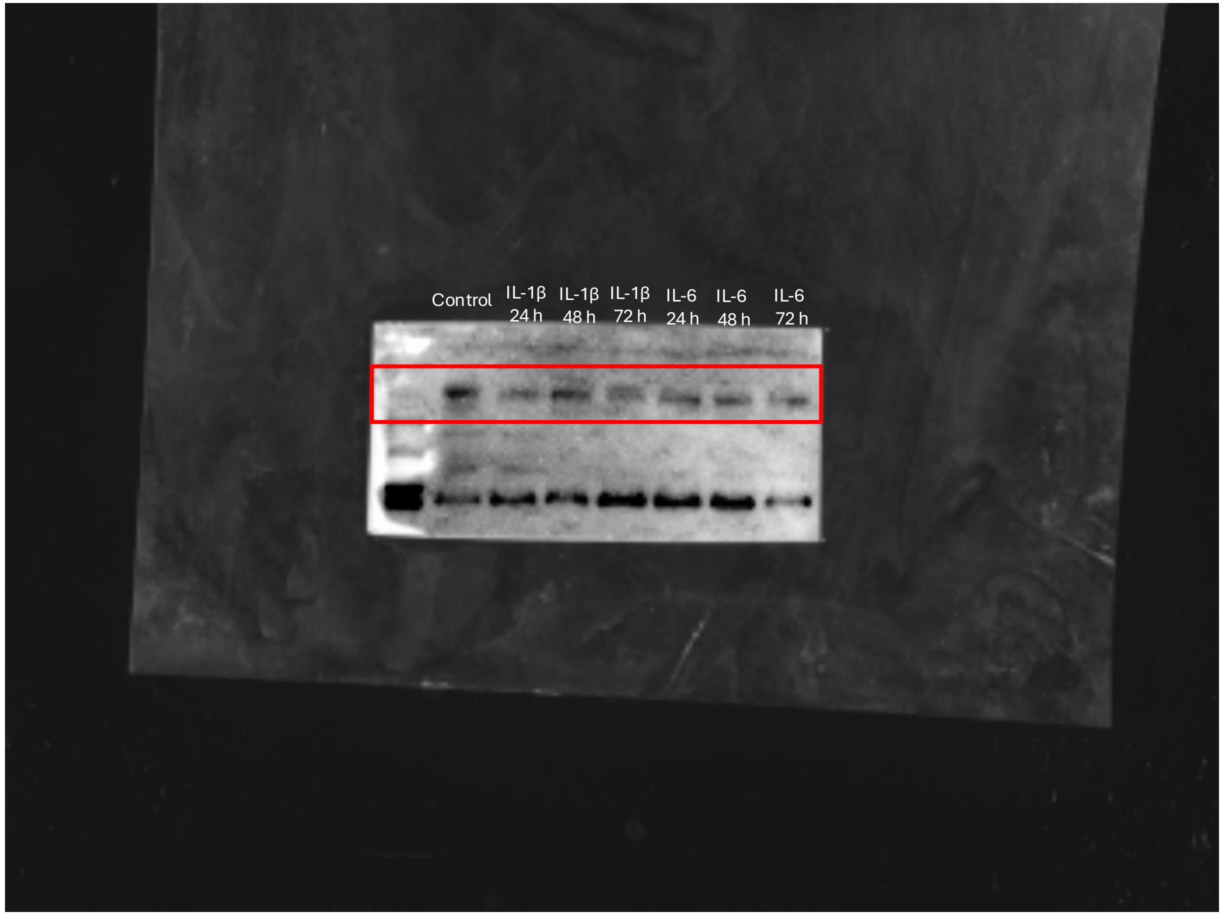

β-actin blot (uncropped)

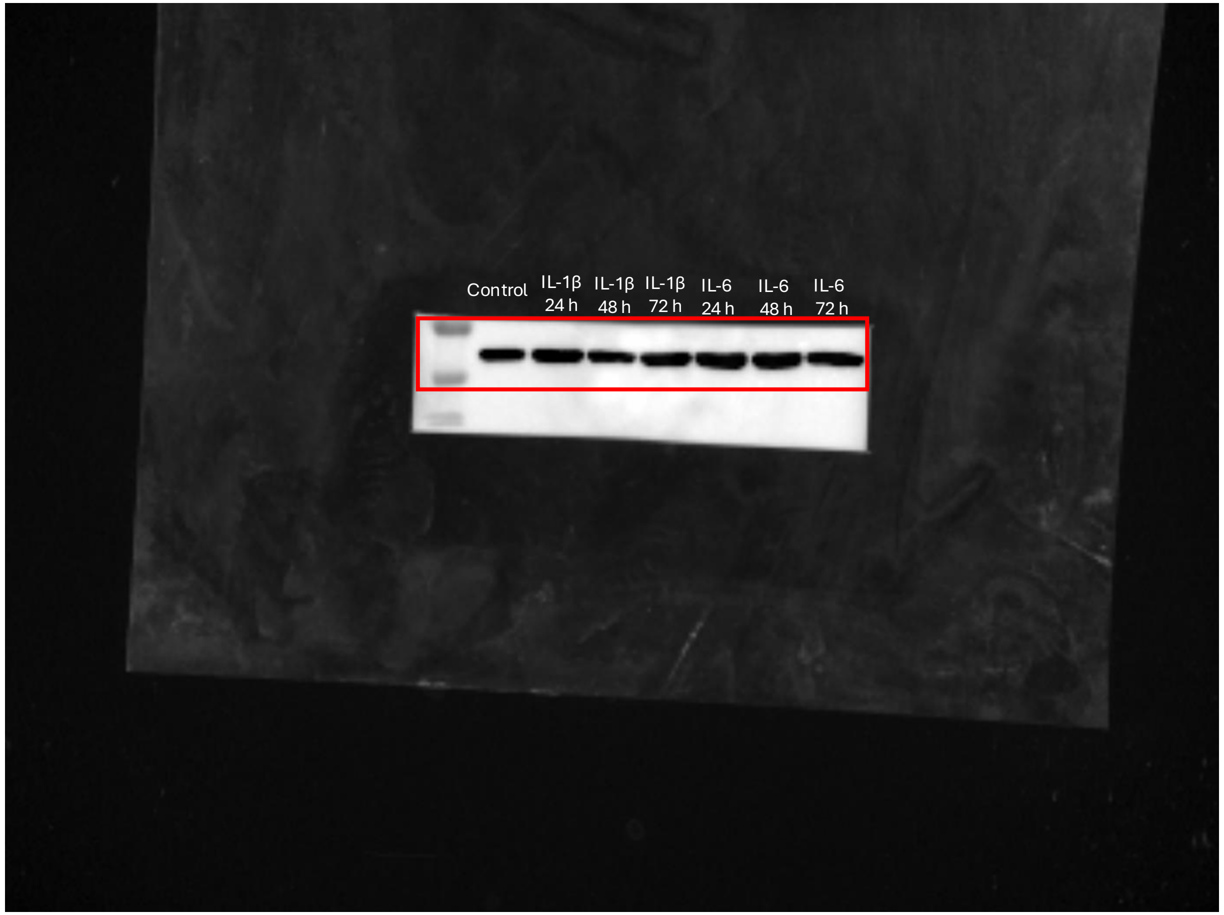

- ABCC5 (uncropped membrane). The anti-ABCC5 polyclonal antibody detected two major immunoreactive bands at approximately 250 kDa and ~80 kDa. The higher molecular weight band (~250 kDa), consistent with the reported molecular mass of full-length ABCC5 (~220–250 kDa), was used for quantification in this study. The lower molecular weight band may reflect non-specific binding or antibody cross-reactivity, which has been reported for some ABCC-family antibodies. Accordingly, only the higher molecular weight band was quantified.

- β-actin (corresponding loading control)

Supplementary Figure S1F – Uncropped ABCC5 and β-actin blots (corresponds to Figure 4K)

ABCC5 blot (uncropped)

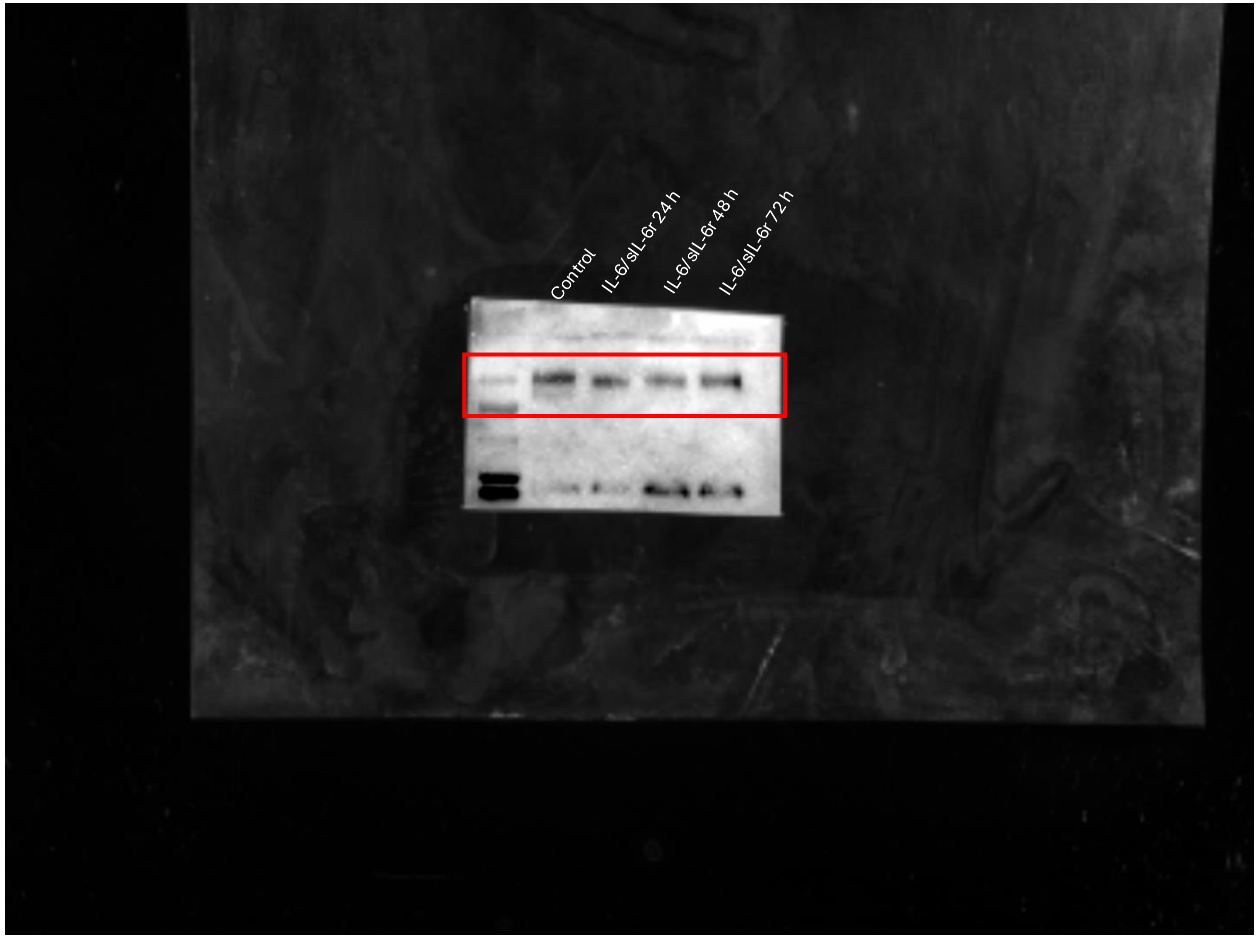

- ABCC5 (uncropped membrane). The anti-ABCC5 polyclonal antibody detected two major immunoreactive bands at approximately 250 kDa and ~80 kDa. The higher molecular weight band (~250 kDa), consistent with the reported molecular mass of full-length ABCC5 (~220–250 kDa), was used for quantification in this study. The lower molecular weight band likely reflects non-specific binding or antibody cross-reactivity, which has been reported for some ABCC-family antibodies. Accordingly, only the higher molecular weight band was quantified.

β-actin blot (uncropped)

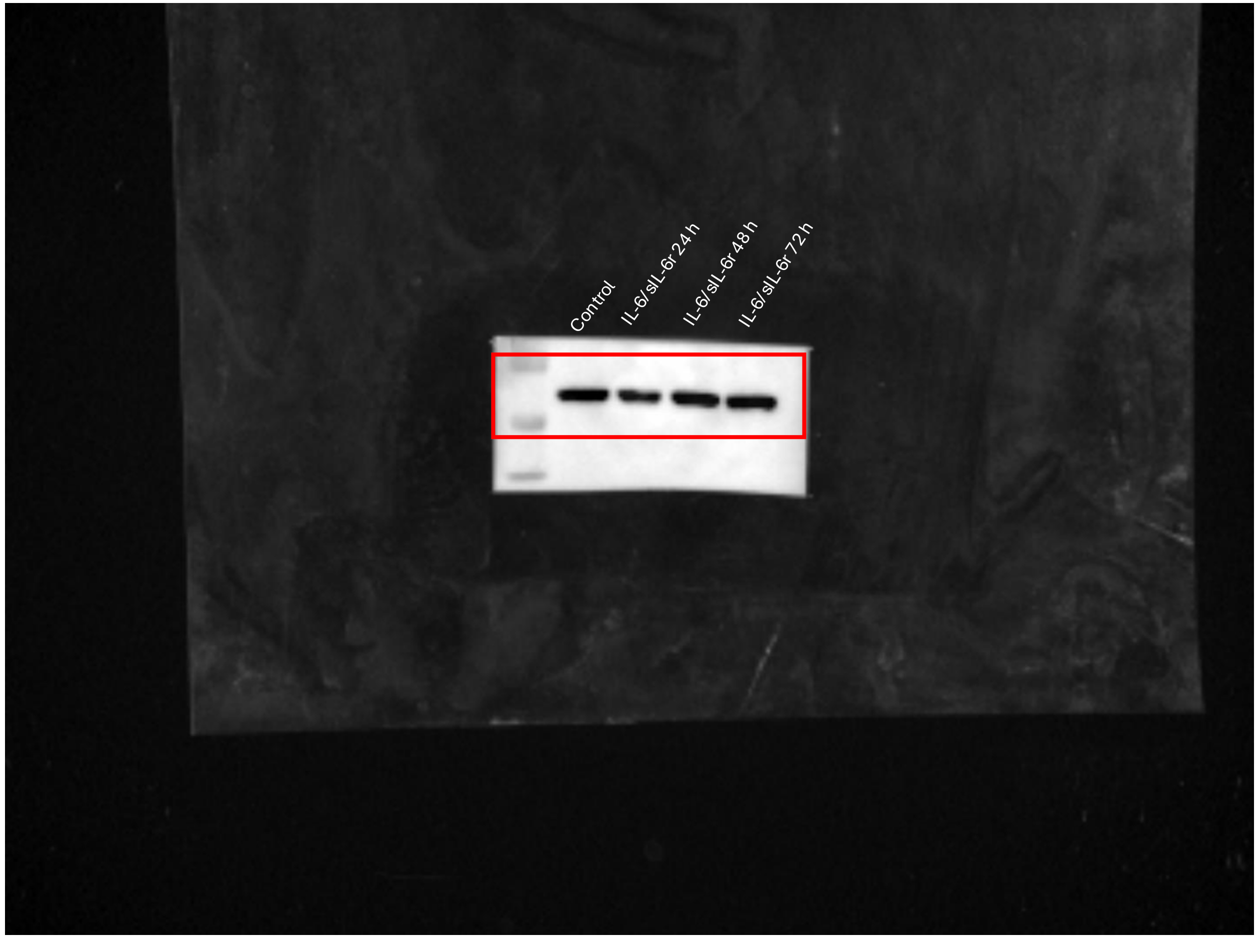

- β-actin (corresponding loading control)
